# Implicitly and explicitly encoded features can guide attention in free viewing

**DOI:** 10.1167/jov.20.6.8

**Published:** 2020-06-12

**Authors:** Aoqi Li, Jeremy M. Wolfe, Zhenzhong Chen

**Affiliations:** School of Remote Sensing and Information Engineering, Wuhan University, Wuhan, PR China; Brigham & Women's Hospital, Cambridge, MA, USA; Harvard Medical School, Boston, MA, USA

**Keywords:** priming, top-down, free viewing, attention

## Abstract

It is well known that priming, probably by the contents of working memory, can influence subsequent visual task performance. How ubiquitous is this effect? Can incidental exposure to visual stimuli influence the deployment of attention when there is no explicit visual task? Results of two experiments show that a preceding stimulus can influence free-viewing eye movements. A simple change detection task was used as the cover task. The initial memory display was the priming display, while subsequent filler display constituted the free-viewing display of our interest. In Experiment 1, subjects were asked to memorize the number of items in the priming display. Subjects were not explicitly instructed to attend to features, but these might still be implicitly encoded. In Experiment 2, a more complex change detection task required subjects to memorize the number, color, and shape of priming items. Here, prime features were attended and, presumably, explicitly encoded. We were interested to know whether incidentally or explicitly encoded features of prime items would influence attention distribution in the filler display. In both experiments, items sharing color and shape with the prime were attended more often than predicted by chance. Items sharing neither color nor shape were attended less often. Items sharing either color or shape (not both) could also attract attention showing that the priming need not be based on a bound representation of the primed item. Effects were stronger in Experiment 2. No intention or top-down control appears to be needed to produce this priming.

## Introduction

How do the contents of your mind influence what you look at in the world? Obviously, holding a specific goal in mind can drive visual behavior. If you want to see a bird, your eye movements will be shaped by that intention. But suppose that the current visual scene is unrelated to what you have in mind. Will your gaze still be influenced by your thoughts? Imagine that you are walking down the street, thinking about tomatoes that are in your kitchen. As you are freely viewing the world, will your gaze be drawn to items that are red and/or round because you are thinking about something red and round? The two experiments, presented here, suggest that the answer would be “yes.”

This question of the influence on visual behavior of irrelevant stimuli held in memory has been addressed previously. A color, held in memory, will bias eye movements in a visual search task, even if the color is totally irrelevant to the task (Soto et al., 2006, 2008). The recent history of visual search will shape performance on the next visual search task, even if the history is not relevant (Failing & Theeuwes, 2018; Hillstrom, 2000; Kristjánsson & Campana, 2010; Maljkovic & Nakayama, 1994). But these are influences of recent past on a specific task. We are asking if there are similar influences when the observer is simply “free viewing.” Free viewing is a somewhat ambiguous concept. Presumably, experiments that involve free viewing are trying to capture the state in which your eyes are open but where you are not doing an explicit visual task (perhaps, navigating as you stroll down the street looking for nothing in particular). In the lab, some free-viewing studies involve quite an explicit task during the free-viewing period of the experiment, for example, memorizing images or performing a change detection task (Pertzov et al., 2009). In other studies, subjects were given minimal instructions, for example, “watch and enjoy the video clips, try to pay attention, but don’t worry about small details” (Berg et al., 2009). However, even an instruction to just “look at the pictures” (Hooge et al., 2005) imposes some sort of task, especially when the stimuli are present for many seconds. Are you really “free viewing” if you are constrained to look at a static image for 30 seconds?

We do not mean to suggest that there is anything wrong with the free-viewing studies we mention above. However, we were seeking specifically for effects of the contents of the mind on the deployment of the eyes over a stimulus that we did not discuss with the participants. Accordingly, from the vantage point of our observers, our free-viewing stimuli were merely the filler periods between task-relevant stimuli. Observers performed a change detection task. During the interval between the two frames of that task, visual things happened on the screen but we did not tell people anything about them. We simply tracked their eyes, looking for and finding evidence that the initial memory encoding display of the change detection task influenced their free viewing of this time-filling, filler display. Our paradigm has some similarity to the classic contingent capture paradigm of Folk et al. (1992), in which subjects were presented with an irrelevant precue before the target display. That precue could then influence processing of the target display. The main difference between our proposed paradigm and Folk's is that in our study, observers are not given any task or instruction with regards to the critical filler display after the memory display, while in Folk's experiment, observers respond to a target in the critical display after the cue. These are two different ways to look at the processing of seemingly incidental stimuli.

Much of the evidence for the effects of prior stimulation on current selective attention is based on studies of visual search and related tasks. Specifically, performance on the current search is often influenced by previous behavior. Even in efficient search tasks, where participants can quickly identify and select the target, responses are influenced by prior trials (Maljkovic & Nakayama, 1994, 1996). When targets repeat on two consecutive trials, participants respond more quickly than when targets switch. The first trial “primes” the response on the second. Visual search tasks only require observers to search for one instance of one target. However, many real-world search tasks are much more complex than the standard visual search paradigm used in lab experiments. In “foraging” tasks, there are multiple instances of the target in the visual display (Gilchrist et al., 2001; Kristjánsson et al., 2014; Wolfe, 2013). In “hybrid search” tasks, there is more than one type of target, held in the observer's memory (Wolfe, 2012). To better investigate the interaction between visual and memory search, Wolfe et al. (2016) introduced hybrid foraging, in which observers search for multiple instances of several types of targets. As observers collect targets in a hybrid foraging task, there are clear effects of the previous target on the selection of the next. Observers do not choose the next item at random. It is more likely that the next target type will be the same as the preceding (Wolfe et al., 2019). At least this is true when targets are distinguished from distractors by basic features like color and shape. This can be seen as evidence for feature priming in hybrid foraging.

Many spatial attention models assume that locations are prioritized jointly by bottom-up saliency based on local feature contrast and top-down control based on task relevance (Wolfe et al., 1989; Borji & Itti, 2013; Hinde et al., 2017). As noted, attention can also be directed by priming effects, an involuntary bias of spatial attention toward previously selected items (Awh et al., 2012). However, the necessary and sufficient conditions for priming remain unclear. Some argue that the mere exposure to stimulus is enough to induce priming (Theeuwes & van der Burg, 2011). Single-cell studies have shown that responses of neurons in the frontal eye field (FEF) are changed by stimulus repetition (Bichot Schall, 2002). Since the FEF is a candidate for a neural substrate of saliency map (Thompson & Bichot, 2005), if priming sharpens the cortical representation of the repeated stimulus, that may render the stimulus as effectively more salient, compared to its surroundings.

Theeuwes and van der Burg (2013) showed that an irrelevant priming stimulus could alter judgments of temporal order as assessed by either the traditional temporal order judgment task (TOJ) or a simultaneity judgment task (SJ). In the TOJ task, subjects had to answer which of two stimuli occurred first, given different delays between those two stimuli. In the SJ task, subjects were instructed to report whether two stimuli appeared at the same time. Before test trials, a non informative stimulus that was completely irrelevant to the task was shown as the prime. The judgment of initial attentional deployment is based on the prior entry effect, which indicates that the processing of first attended stimulus is accelerated so the time between its physical onset and further processing is reduced. Therefore, if priming indeed makes a stimulus more salient, it should attract attention first and be perceived earlier than a stimulus that is not primed. Experiments demonstrate that the initial deployment of attention was directed to the primed stimulus, causing its prior entry into awareness, despite the fact that subjects were not instructed to search for any target in either task. The mere processing of the non informative prime can lead to the automatic selection of primed stimulus, which is independent of top-down selection.

In contrast, some argue that observers have to do more rather than merely passively view a priming stimulus (Kristjánsson et al., 2013). In one experiment designed to determine the minimum processing required for priming, researchers distinguished between active search and passive viewing trials. It was found that displays requiring no search resulted in no priming. This seems to contradict the idea that priming is automatic and favors a more controlled, top-down account. But in another study that investigated the role of motor response in priming, researchers (Yashar et al., 2013) showed that priming occurred even when the previous trial was a no-go trial in which participants only passively viewed the display, although the effect was larger after a go trial in which they were asked to respond to the target. One possible explanation for the lack of priming in Kristjánsson's study and the smaller effect in Yashar's is that the effects of priming were counterbalanced by the effects of task switching. It has been demonstrated that responses are slower when the previous trial is a no-go trial than when the previous trial is a go trial (Schuch & Koch, 2003; Los & der Burg, 2010). Therefore, it is possible that the task-switching from a passive viewing trial to an active search trial slows responses while no slowing would occur on the second of two active trials, leading to the illusion that active attentional processing is a prerequisite for priming.

Another study arguing against the hypothesis of entirely automatic, bottom-up priming involves two different tasks within a single sequence of stimuli (Yashar et al., 2017). There were trials containing singleton displays related to a search task and probe displays related to a probe task. In the search task trials, subjects were asked to discriminate the orientation of the target feature singleton. The probe task trials required subjects to discriminate the orientation of the probe with singleton displays now being irrelevant to the task. Trial type switched after every two trials, so there were four types of trial pairs: (1) search, search; (2) probe, probe; (3) probe, search; and (4) search, probe. The authors found that priming only clearly occurred in the first condition, where singletons were related to the task in both trials, and weakly in the third condition, where singletons were task related in the second trial, casting doubt on previous findings that stimulus repetition alone automatically makes the primed stimulus more salient, regardless of what observers intend to do. However, in this experiment, no singleton appeared on probe displays. So it is hard to say whether priming would emerge if singletons and probes were combined in one display.

In the present study, we use a different method to investigate whether task-related factors are necessary in priming. To eliminate the influence of top-down control, we tried to create a test stimulus that appears to the observer to be nothing more than the object of free viewing, which is different from other designs involved with switching tasks. To this end, we designed a task where observers would think that the critical, freely viewed stimuli were just fillers between the memory display and the test display of a change detection task. We show that features and objects found in the memory display do exert an influence on selective attention and eye movements during this free viewing of the filler.

## Experiment 1

### Participants

Twelve participants (seven females, average age 29) from the Brigham and Womens Hospital Visual Attention Lab volunteer pool were tested. All had normal or corrected-to-normal vision and were naive to the purpose of our experiment. Participants were paid $11/hour and gave informed consent approved by the Brigham and Womens Hospital Institutional Review Board.

### Apparatus and stimuli

**Figure 1.**
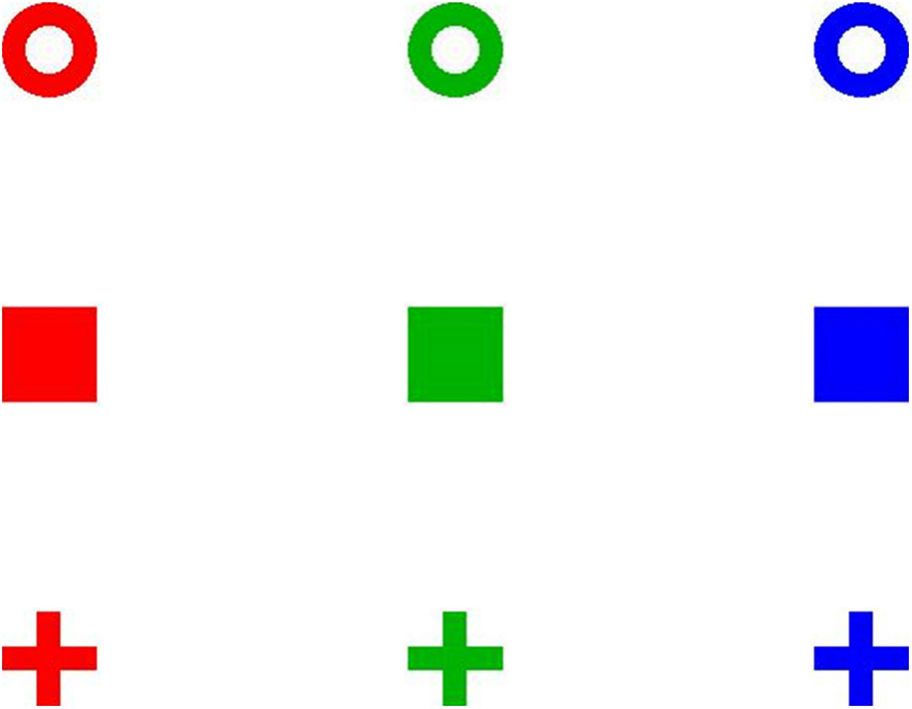
The nine possible item types.

Eye movements were recorded by the Eyelink 1,000 system (SR Research Ltd, Ontario, Canada) with sample rate of 1,000 Hz. The accuracy of the Eyelink 1,000 system can be as good as 0.15^○^ with 0.25^○^ to 0.5^○^ accuracy typical, and the precision is smaller than 0.01^○^ root mean square (RMS), according to its user manual. A 9 point calibration and validation was conducted before each experiment. The online parser incorporated in the Eyelink 1,000 system was used to analyze eye position data into meaningful events and states (saccades and fixations). Experiments were run using MATLAB 8.3 with Psychtoolbox. Stimuli were presented on a 24-in. Dell 2407WFP REV A04 monitor, with a screen resolution of 1,024 × 768 and a refresh rate of 60 Hz. Participants sat with their heads immobilized in a chin rest at 65 cm from the screen. Items with a size of 1.52^○^ × 1.52^○^ were placed on a white background. Items were placed within an area of about 26^○^ × 20^○^. Item colors could be red, green, and blue. Possible shapes were an open circle, a solid square, and a plus. Thus, there were 3 × 3 = 9 possible types of item, as shown in Figure 1.

### Procedure

**Figure 2.**
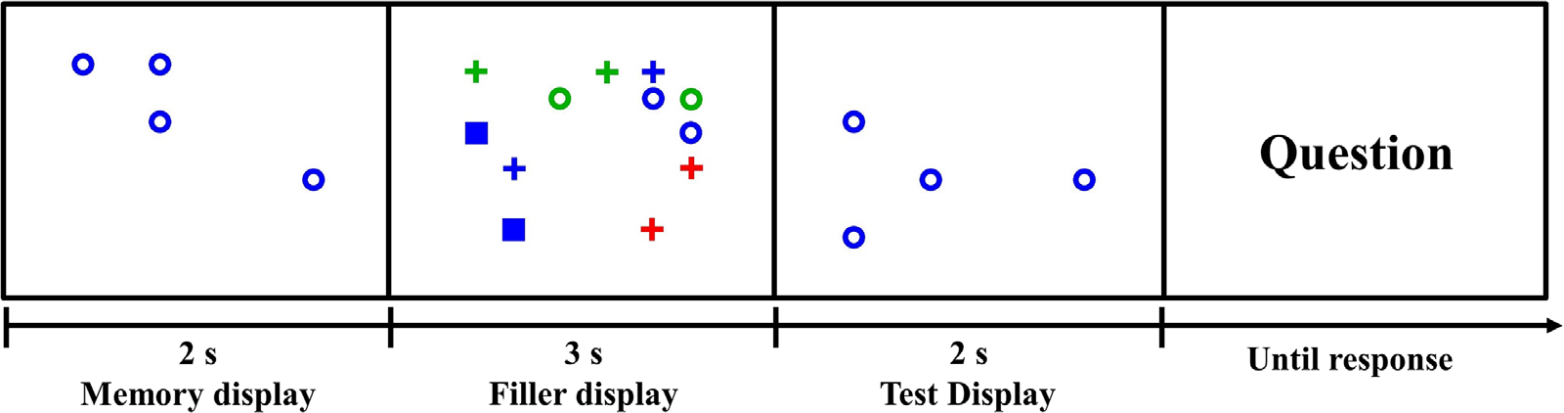
Example of a prime-present trial in Experiment 1. Observers had to answer whether the number of items (blue circles) changed. Items on the filler display were moving, and eye-tracking data during the in-between period were collected. Positions of all items were randomly generated to eliminate any spatial bias. Chance levels in this trial are full match (2/12), color match (4/12), shape match (2/12), and no match (4/12).

Figure 2 shows the sequence of a trial. Participants were told that they were participating in a change detection task, in which they would see a memory display and a test display, separated by a few seconds, and would need to report differences between those displays. This change detection task was only a cover task that was not the actual subject of our interest. We were interested in participants’ eye movements over the filler display during a free-viewing period between the memory display and the test display of the change detection task. Items in the memory display and the test display were defined by their color and shape. On a given trial, all items in the memory display and the test display were of the same color and shape. A static set of items, henceforth called the priming stimuli, was shown in the memory display. This memory display was shown for 2 s.

Between the memory display and the test display, observers saw 12 items that moved at a fixed speed in a pseudorandom manner. The set size of 12 items was chosen to seem like a plausible distraction while not being too congested to permit clear eye tracking. We used dynamic rather than static filler displays because we had found in a pilot study that subjects tended not to move their eyes if the filler display was static. They simply fixated near the center of the display. Moving displays induced observers to move their eyes. An example of the initial state of a filler display is shown in the second panel of Figure 2. These 12 items consisted of two copies of six of the nine possible combinations of color and shape, that is, green circle, blue circle, blue square, red plus, green plus, and blue plus. If there is only one item of each kind, it may fail to capture visual attention due to its initial position and moving direction. For example, if the relevant item happened to be far away from where a subject was initially looking and that item happened to be moving farther away, it might be ignored during the whole trial. The probability that both instances of a type of item would be in randomly bad positions is smaller, so we decided to use two copies of each type of item. There were $C_{9}^{6}=84$ ways of selecting six out of the nine items in Figure 1, so there were 84 variations of the filler display with regard to item color and shape. The color or shape of the priming stimulus was always represented in the moving display. The specific combination of color and shape, used in the memory display, was present in the moving filler display on about 50% of trials. These were “prime-present” trials. The other trials were “prime absent.” Figure 2 represents a prime-present trial because open blue circles, the priming stimuli in the memory display, are present in the moving, filler display.

Participants were not given any explicit instructions about the moving, filler display. As far as they knew, it was mere filler between the memory display and the test display of the change detection task. As a free-viewing display, we think that this is an improvement over designs where participants are told to just look at a display. Here visual stimuli are just present, as they might be while you are walking down the street, with your eyes open but thinking about something else. After a 3-s free-viewing period, the test display of the change detection task appeared for 2 s. The shape and color of items in the test display were the same as that of items in the memory display, but the number and/or position of items might change. When the question appeared, participants had to respond by key press to indicate whether the number of items changed. Response time was unlimited. Feedback was given to participants indicating whether the response was correct.

Experiment 1 consisted of two blocks with 90 trials each. At the beginning of the first block, there were 5 practice trials. If the correct response rate was below 80%, participants had to perform another 5 practice trials. This process repeated until the accuracy reached or exceeded 80%.

### Data analysis

Eye-tracking data during the free-viewing period were analyzed to investigate whether the priming stimuli influence subsequent eye movements. Items in the filler display were moving, but we could obtain coordinates indicating where subjects were looking on each frame. Both sample eye positions and calculated fixations were used. Sample eye positions are the data directly recorded by the eye tracker. Sample eye position is useful because, with moving stimuli, it could be that the eyes are tracking an item over space. These pursuit eye movements would not register as fixations but would represent a type of “fixation” for our purposes. Eye position data can also be classified into fixations and saccades by the online parser incorporated in the Eyelink 1000 system.

We used the following protocol to analyze the eye movement data. First, we located the eye position (eye position analysis) or fixation (fixation analysis) on each frame of the filler display. The refresh rate of the monitor was 60 Hz. The sampling rate of the Eyelink 1000 system was 1,000 Hz, so up to 1,000 eye positions could be recorded in 1 s. Thus, on each frame, there could be 16 recorded eye positions. These position measures would be very similar. We used the first one in each frame for the following analyses. Fixations lasted more than 1/60 s, so on each frame, there was at most one fixation. Only frames containing fixations were used for fixation analysis.

For each frame, we then found the item that was the closest to the sample eye position or fixation, using the Euclidean distance between eye positions (or fixations) and item centers. That item was defined as the attended item, recognizing that this was an imperfect measure because objects were moving and there was no guarantee that the closest item was the attended target. Note, however, that errors in assigning fixation/attention to the correct item are only going to degrade the data. Such errors will not produce spurious bias toward items sharing features of the prime stimulus.

The attended items were compared to prime stimulus and classified into one of four categories: (1) full match: attended item shared both color and shape with the prime stimulus; (2) color match: the item shared the color but not the shape of the prime; (3) shape match: the item shared the shape but not the color of the prime; and (4) no match: the item shared neither the shape nor the color of the prime.

After the classification, we obtained the number of frames belonging to each category during the trial. These were converted to percentages to create the distribution of the probabilities of each of the four categories for each trial. At the same time, chance levels of the four categories were calculated for each trial. For example, in Figure 2, chance level of full match was 2/12 (2 blue circles out of the 12 items), of color match was 4/12 (2 blue squares and 2 blue pluses), of shape match was 2/12 (2 green circles), and of no match was 4/12 (2 red pluses and 2 green pluses).

Finally, the probabilities and chance levels for each category from all the trials were averaged for each subject and compared by standard statistical methods. Chance levels for each trial are determined by which six of nine possible items are selected for that trial's filler display. On prime-present trials, we need to make sure that there is a full match, so the probability of full match is constantly 1/6. Then the remaining five items in the filler display are randomly selected from eight items (apart from the prime). The probability of selecting a no match (4/8) is twice the probability of color match (2/8) and shape match (2/8). Therefore, the probability of shape match in the display is equal to the probability of color match, which is (1 − 1/6) * (2/8) = 5/24 and the probability of no match is (1 − 1/6) * (4/8) = 5/12. On prime-absent trials, there is no full match, so the probability of shape match or color match is 2/8 = 1/4 each, and the probability of no match is 4/8 = 1/2. Since we do not know which six items were randomly selected in each trial, chance level varies from trial to trial, but the average chance levels should approximate this calculation.

### Results

#### Change detection task

The average accuracy was 93% for prime-present and 93% for prime-absent trials.

#### Eye position analysis

**Figure 3.**
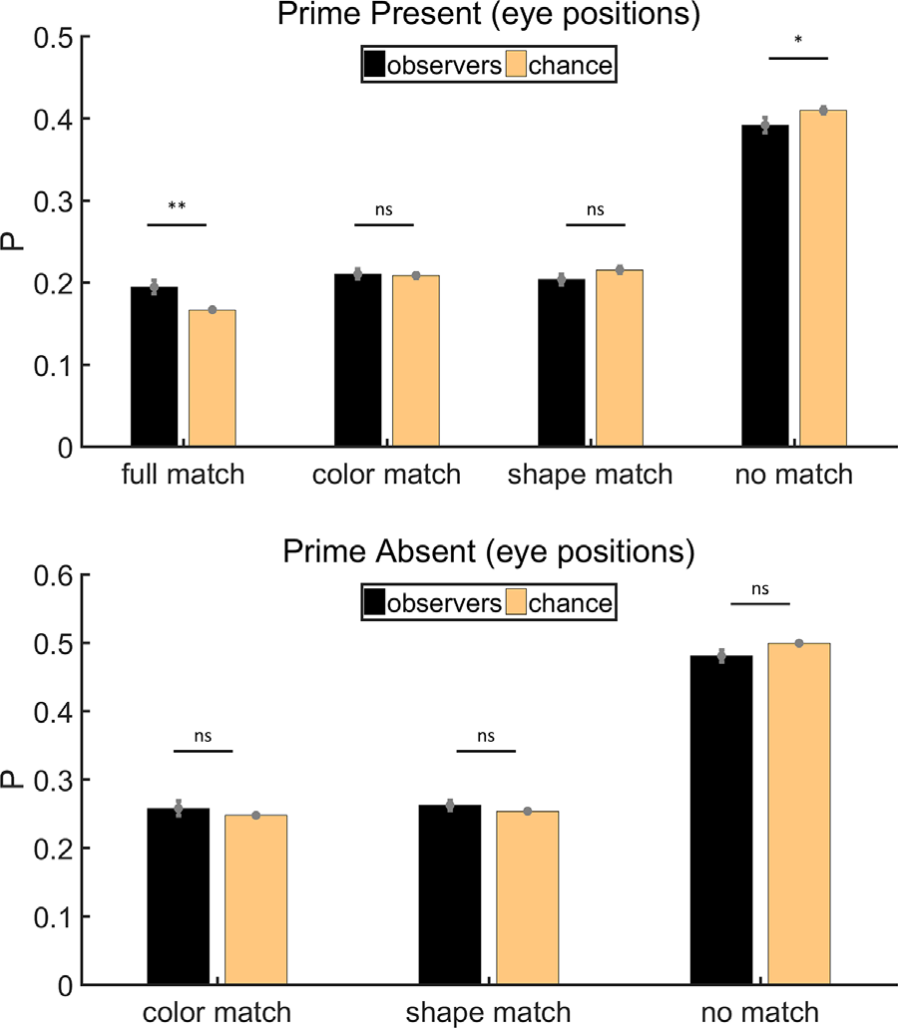
Results of eye position analysis for Experiment 1. Full match: attended item shared color and shape with the prime stimulus; color match: they shared the same color but had different shapes; shape match: they had different colors but shared the same shape; no match: they had different colors and different shapes. Error bars are ± 1 standard error. *p < 0.05. **p < 0.01.

Trials in which we could not collect any eye-tracking data were excluded before the calculation (∼0.4%). As mentioned in the Procedure, chance level varies with the choice of items. Therefore, chance is a little different for each observer, but the average matches our calculation in the Procedure. Variations in chance as shown by error bars in Figure 3 were still taken into account when we did the statistical analysis. Instead of using a one sample test against chance level, we regarded the chance as another sample and used a paired *t* test. Since our data were proportions obtained from a count, arcsine transformation was used to transform them to make them more suitable for statistical analysis. Analyses were done on transformed data, but figures were drawn with untransformed data. Figure 3 shows the results of eye position analysis. On prime-present trials, the percentage of items in no match category was below chance (paired *t* test, *t*(11) = −2.92, *p* < 0.05, Cohen's *d* = −0.84), demonstrating there was some priming effect during the free-viewing period. The probability that attended items matched the prime color and shape was above chance level (paired *t* test, *t*(11) = 3.46, *p* < 0.01, Cohen's *d* = 1.00). There was no significant deviation from chance for the probabilities of attending items that matched only the color (paired *t* test, *t*(11) = 0.23, *p* = 0.82, Cohen's *d* = 0.07) or only the shape (paired *t* test, *t*(11) = −1.81, *p* = 0.10, Cohen's *d* = −0.52), suggesting that the whole item served as a prime rather than the component features. On prime-absent trials, evidence for priming is weak. Items with neither the prime color nor the prime shape were attended less often than predicted by chance, but this was marginally significant (paired *t* test, *t*(11) = −2.18, *p* = 0.0516, Cohen's *d* = −0.63). Neither color or shape had any significant advantage in drawing the eyes, with probabilities about chance level (paired *t* tests, color: *t*(11) = 0.96, *p* = 0.36, Cohen's *d* = 0.28; shape: *t*(11) = 1.32, *p* = 0.21, Cohen's *d* = 0.38).

#### Fixation analysis

**Figure 4.**
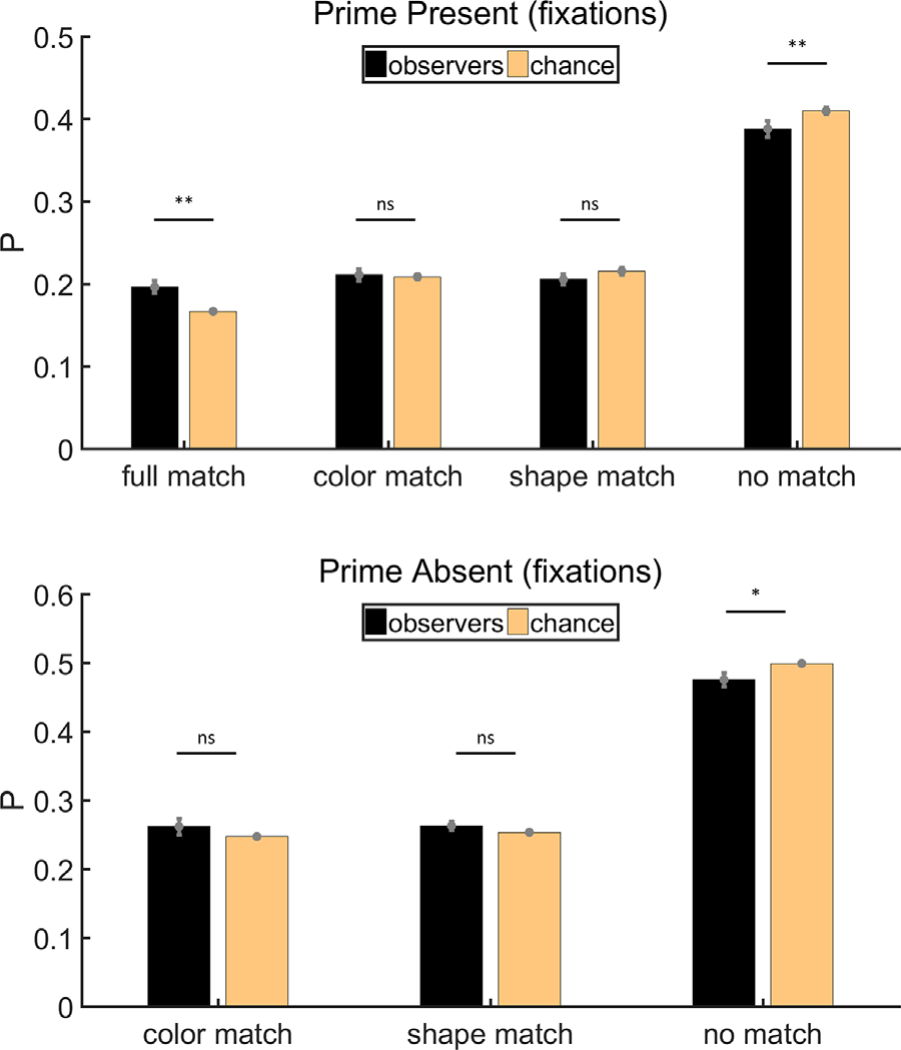
Results of fixation analysis for Experiment 1. Full match: attended item shared color and shape with the prime stimulus; color match: they shared the same color but had different shapes; shape match: they had different colors but shared the same shape; no match: they had different colors and different shapes. Error bars are ± 1 standard error. *p < 0.05. **p < 0.01.

We repeated the analysis using fixations, rather than eye positions. Trials in which we could not get any fixations were excluded (∼0.4%). On both prime-present and prime-absent trials, the average number of fixations is 7. Figure 4 shows the results of fixation analysis. On prime-present trials, the probability of fixated items being classified as no match was below chance level (paired *t* test, *t*(11) = −3.21, *p* < 0.01, Cohen's *d* = −0.93) while the probability of items in full match was above chance level (paired *t* test, *t*(11) = 4.09, *p* < 0.01, Cohen's *d* = 1.18). The probabilities of attended items belonging to the remaining two categories were near chance level (paired *t* tests, color: *t*(11) = 0.25, *p* = 0.808, Cohen's *d* = 0.07; shape: *t*(11) = −1.46, *p* = 0.171, Cohen's *d* = −0.42, respectively). This is similar to the results of eye position analysis, showing that there was some priming effect on prime-present trials, and such effect emerged under the joint influence of color and shape rather than one of them. On prime-absent trials, the ratio of items in no match was below chance (paired *t* test, *t*(11) = −2.35, *p* < 0.05, Cohen's *d* = −0.68), but the ratios of items in the other two categories were not significantly above chance (paired *t* tests, color: *t*(11) = 1.29, *p* = 0.222, Cohen's *d* = 0.37; shape: *t*(11) = 1.47, *p* = 0.170, Cohen's *d* = 0.42, respectively). Hence, the priming effect was less pronounced on prime-absent trials.

##### The *N*th fixations

**Figure 5.**
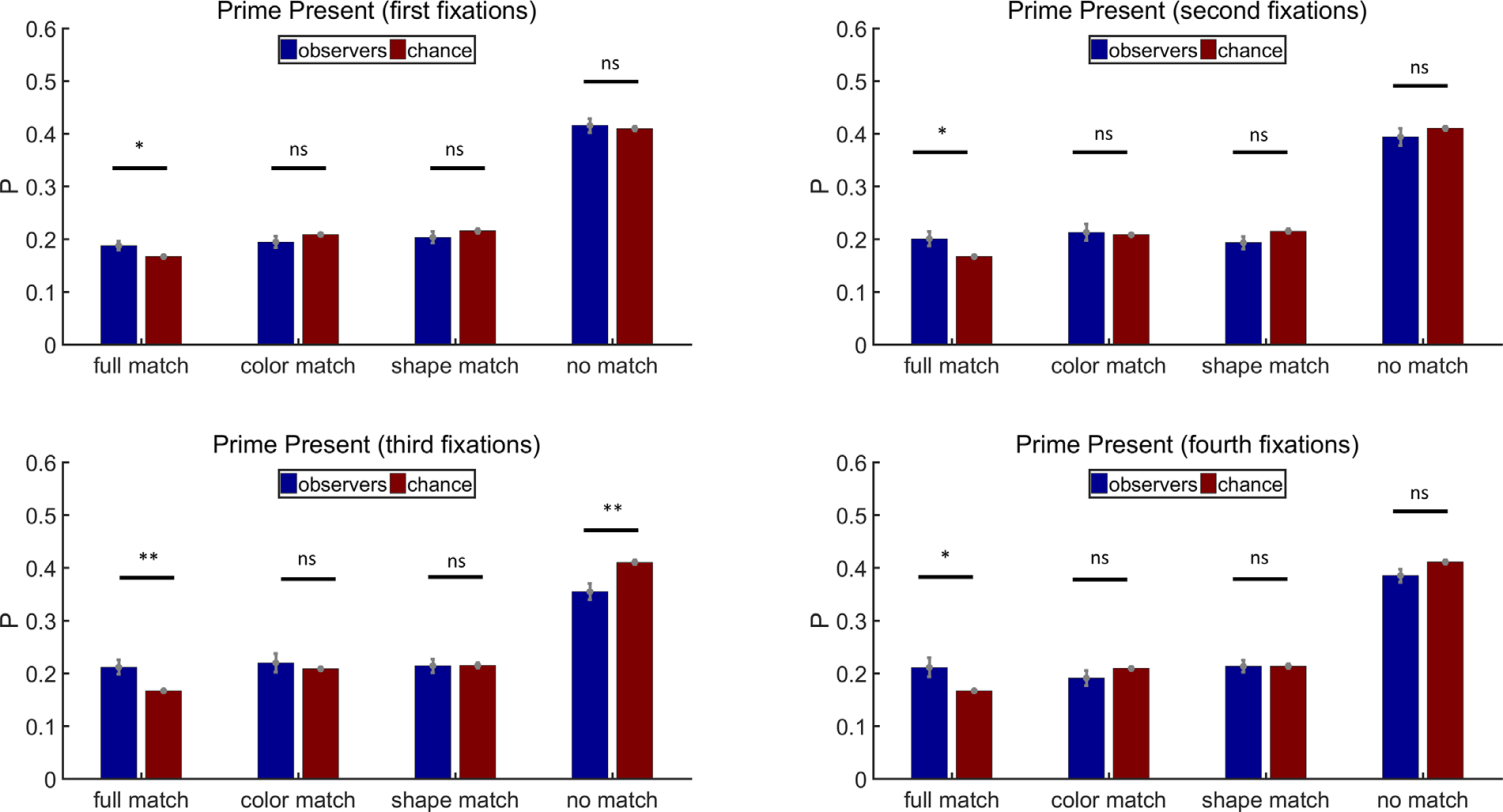
Results of the first, second, third, and fourth fixation analysis for prime-present trials in Experiment 1. Full match: attended item shared color and shape with the prime stimulus; color match: they shared the same color but had different shapes; shape match: they had different colors but shared the same shape; no match: they had different colors and different shapes. Error bars are ± 1 standard error. *p < 0.05. **p < 0.01.

In addition to analyzing the distributions of filler display fixations overall, we analyzed the first, second, third, and fourth fixations, separately. All participants had four-fixation trials. On average, 163 trials per participant were included (max = 180, min = 124) for the analysis. Figure 5 shows the results of prime-present trials in Experiment 1. The probability of the first fixations visiting full match items was above chance (paired *t* test, *t*(11) = 2.45, *p* < 0.05, Cohen's *d* = 0.71), but a single feature of the priming stimuli did not bias the first fixations (paired *t* tests, color match: *t*(11) = −1.32, *p* = 0.21, Cohen's *d* = −0.38; shape match: *t*(11) = −1.18, *p* = 0.26, Cohen's *d* = 0.34; no match: *t*(11) = 0.47, *p* = 0.65, Cohen's *d* = 0.13). The influence of the prime on the second fixations was similar to that on the first fixations (paired *t* tests, full match: *t*(11) = 2.38, *p* < 0.05, Cohen's *d* = 0.69; color match: *t*(11) = 0.13, *p* = 0.90, Cohen's *d* = 0.04; shape match: *t*(11) = −2.00, *p* = 0.07, Cohen's *d* = −0.58; no match: *t*(11) = −1.18, *p* = 0.26, Cohen's *d* = −0.34). The joint influence of both color and shape was the strongest when only all the third fixations were considered (paired *t* tests, full match: *t*(11) = 3.57, *p* < 0.01, Cohen's *d* = 1.03; color match: *t*(11) = 0.50, *p* = 0.63, Cohen's *d* = 0.14; shape match: *t*(11) = −0.15, *p* = 0.88, Cohen's *d* = −0.04; no match: *t*(11) = −3.60, *p* < 0.01, Cohen's *d* = −1.04) but became weaker on the fourth fixations (paired *t* tests, full match: *t*(11) = 2.49, *p* < 0.05, Cohen's *d* = 0.72; color match: *t*(11) = −1.43, *p* = 0.18, Cohen's *d* = −0.41; shape match: *t*(11) = −0.09, *p* = 0.93, Cohen's *d* = −0.03; no match: *t*(11) = −2.1, *p* = 0.06, Cohen's *d* = −0.61).

**Figure 6.**
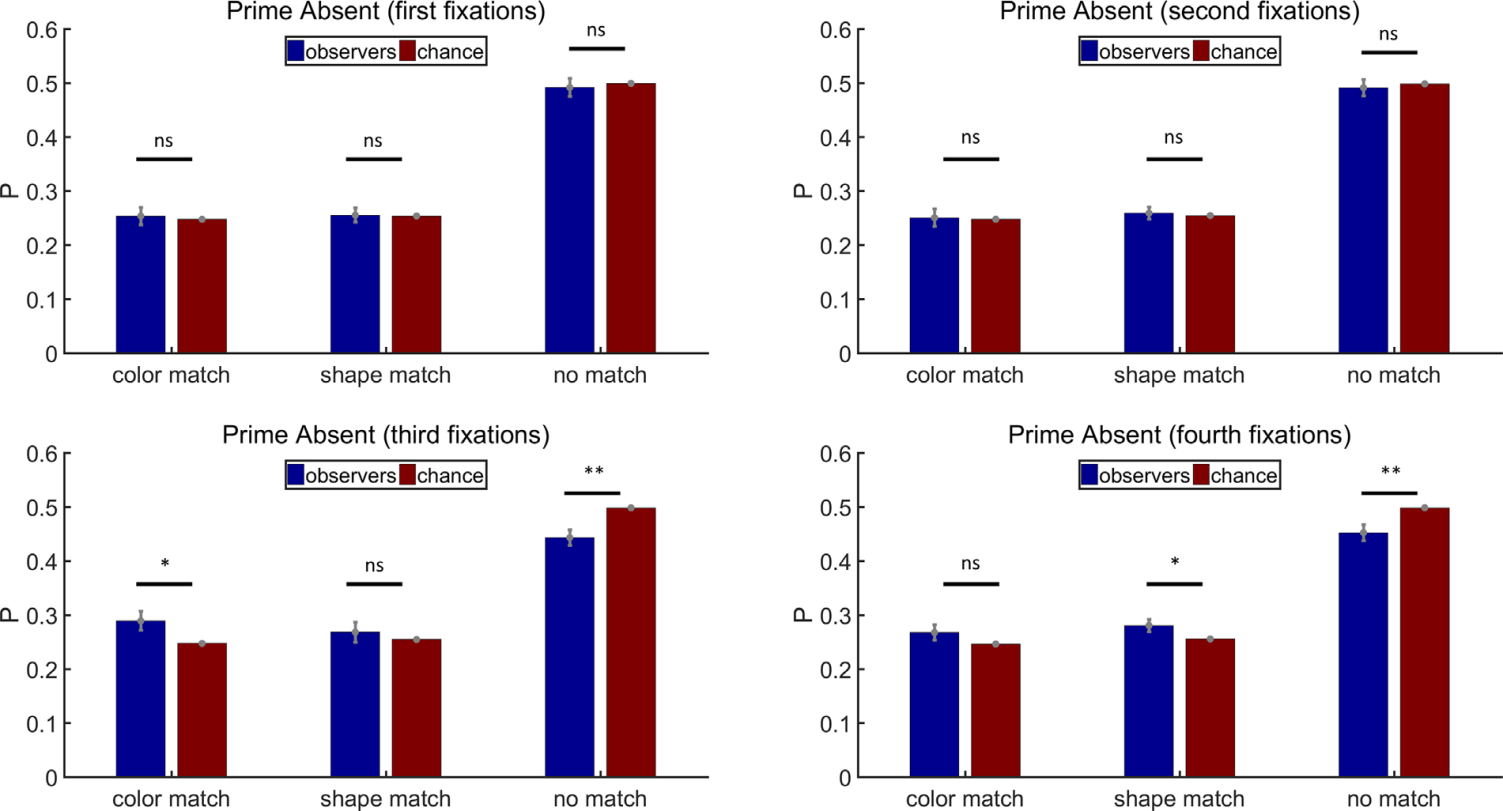
Results of the first, second, third, and fourth fixation analysis for prime-absent trials in Experiment 1. Full match: attended item shared color and shape with the prime stimulus; color match: they shared the same color but had different shapes; shape match: they had different colors but shared the same shape; no match: they had different colors and different shapes. Error bars are ± 1 standard error. *p < 0.05. **p < 0.01.

As shown in Figure 6, on prime-absent trials, the priming stimuli did not have any influence on the first fixations (paired *t* tests, color match: *t*(11) = 0.30, *p* = 0.77, Cohen's *d* = 0.09; shape match: *t*(11) = 0.02, *p* = 0.98, Cohen's *d* = 0.01; no match: *t*(11) = −0.44, *p* = 0.67, Cohen's *d* = −0.13). Neither did they have any influence on the second fixations (paired *t* tests, color match: *t*(11) = 0.05, *p* = 0.96, Cohen's *d* = 0.01; shape match: *t*(11) = 0.32, *p* = 0.76, Cohen's *d* = 0.09; no match: *t*(11) = −0.45, *p* = 0.66, Cohen's *d* = −0.13). However, the third fixations landed more frequently on color match items and less on no match items (paired *t* tests, color match: *t*(11) = 2.46, *p* < 0.05, Cohen's *d* = 0.71; shape match: *t*(11) = 0.62, *p* = 0.55, Cohen's *d* = 0.18; no match: *t*(11) = −3.34, *p* < 0.01, Cohen's *d* = −0.96), while the fourth fixations landed more on shape match items and less on no match items (paired *t* tests, color match: *t*(11) = 1.42, *p* = 0.18, Cohen's *d* = 0.41; shape match: *t*(11) = 2.25, *p* < 0.05, Cohen's *d* = 0.65; no match: *t*(11) = −3.21, *p* < 0.01, Cohen's *d* = −0.93).

#### Discussion

In this experiment, the change detection task only required subjects to memorize the number of priming items before the free-viewing display. Color and shape of memory display items were irrelevant to the change detection task. Nevertheless, those features still served to bias eye movements in a task-irrelevant, free-viewing episode that fell between the memory display and the test display of the change detection task. By analyzing the first through fourth fixations, we found that the effect of the prime seemed to grow after the initial fixations. We did not see a bias toward items that shared only a single feature with the prime. It could be that only the bound combination of color and shape served as the prime. However, when we analyzed individual fixations instead of all fixations during a trial, we found that the influence of color and that of shape did not appear simultaneously. In addition, the effects of each individual feature might be transient or variable, which might explain why single-feature priming was not obvious when all fixations were taken into account. The influence of single features did not appear until the third fixations, later than that for priming by both features.

In Experiment 1, features like color and shape were not only irrelevant to the filler display but also irrelevant to the memory and test displays. Would the results be same if subjects had to explicitly hold these features in their memory? We examine this question in Experiment 2.

## Experiment 2

### Participants

After finishing Experiment 1, the same 12 observers participated in Experiment 2, allowing comparisons within observers.

### Apparatus and stimuli

The apparatus and stimuli described in Experiment 1 was also used in Experiment 2.

### Procedure

**Figure 7.**
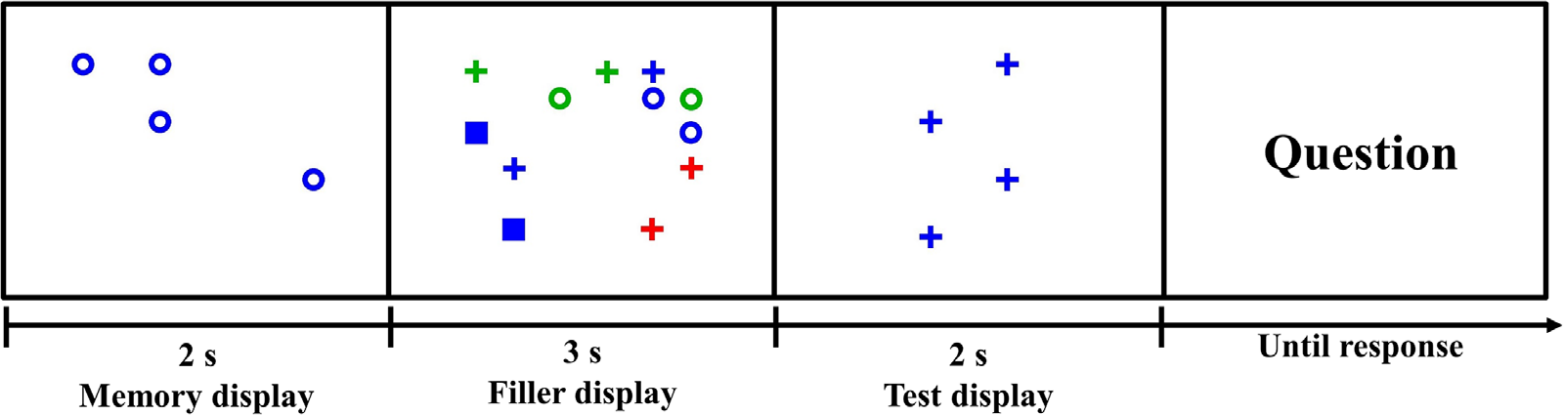
Example of a prime-present trial in Experiment 2. Participants had to choose which of the following aspects of items changed: (1) number, (2) color, (3) shape, or (4) none. In this trial, the correct response would be shape.

Experiment 2 was the same as Experiment 1 with one change to the change detection task. In Experiment 2, the shape or color of items could change between the memory display and the test display, as could the number of items. Participants were asked to identify the nature of the change: color, shape, number, or none. This method was intended to draw more attention to the color and shape of the prime. Note that the free-viewing filler display between the two frames remained the same. It was irrelevant to the participant's task and no instructions were given about it. Figure 7 shows an example trial in Experiment 2.

Like Experiment 1, Experiment 2 consisted of two blocks with 90 trials each. Prime-present and prime-absent trials were randomly intermixed in the experiment, and the ratio of them was roughly 1:1. At the beginning of the first block, as in Experiment 1, participants repeated five trial practice sessions until the accuracy reached or was above 80%.

### Data analysis

The same data analysis methods, used in Experiment 1, were used in Experiment 2.

### Results

#### Change detection task

The average correct response for the change detection task was 91% for prime-present and 91% for prime absent trials.

#### Eye position analysis

**Figure 8.**
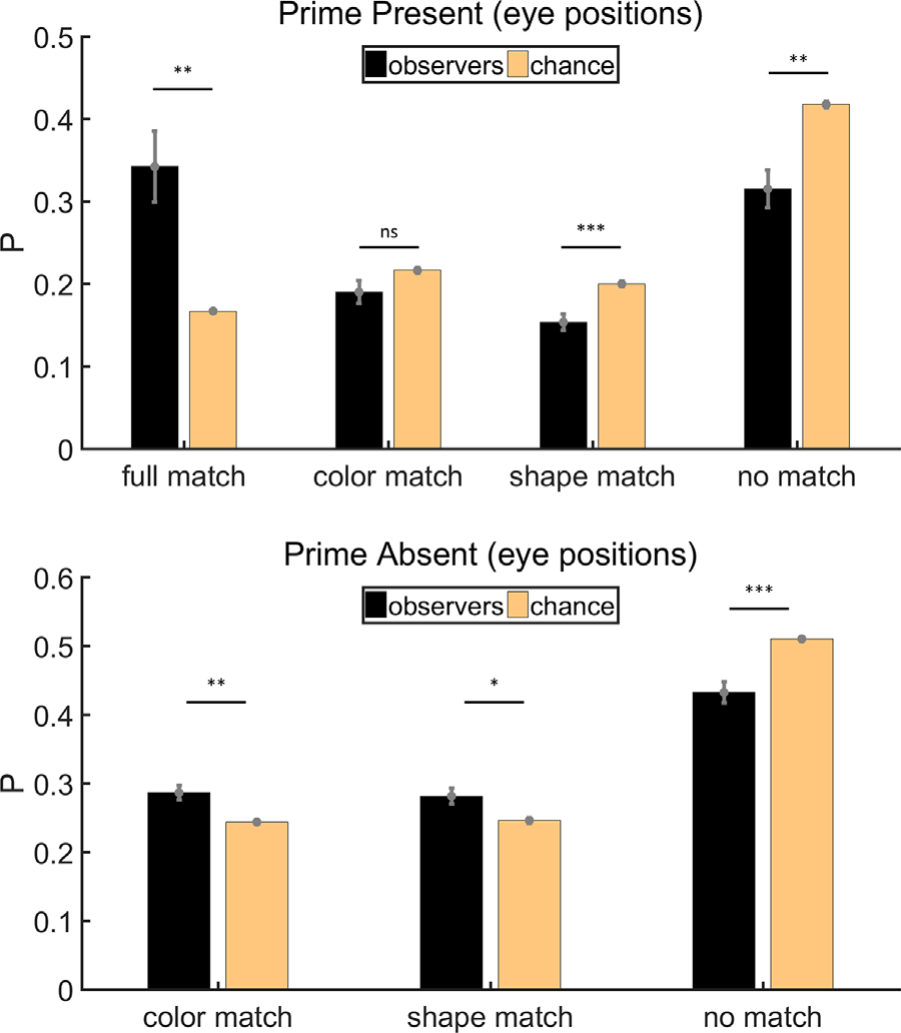
Results of eye position analysis for Experiment 2. Full match: attended item shared color and shape with the prime stimulus; color match: Items shared the same color but had different shapes; shape match: they had different colors but shared the same shape; no match: they had different colors and different shapes. Error bars are ± 1 standard error. *p < 0.05. **p < 0.01. ***p < 0.001.

Trials in which we could not collect any eye-tracking data were excluded before the calculation (∼3%). Figure 8 shows the results of eye position analysis. As can be seen, Experiment 2 produced more robust effects of priming than Experiment 1. On both prime-present and prime-absent trials, the percentage of items in the no match category was below chance (paired *t* tests, prime present: *t*(11) = −4.17, *p* < 0.01, Cohen's *d* = −1.20; prime absent: *t*(11) = −5.07, *p* < 0.001, Cohen's *d* = −1.46). On prime-present trials, the eyes visited full match items more often than chance would predict (paired *t* test, *t*(11) = 4.36, *p* < 0.01, Cohen's *d* = 1.26), demonstrating how color and shape jointly influenced subsequent eye movements. On prime-present trials, the eyes visited color match or shape match items less often than chance would predict (paired *t* tests, color: *t*(11) = −1.85, *p* = 0.09, Cohen's *d* = −0.53; shape: *t*(11) = −5.22, *p* < 0.001, Cohen's *d* = −1.51). This result presumably reflects the zero-sum nature of the task. If there are a large number of fixations on the exact match to the prime, there are necessarily fewer fixations available for the other items. On prime-absent trials, where a perfect match was not possible, the proportions of items in color match and shape match categories were above chance (paired *t* tests, color: *t*(11) = 4.19, *p* < 0.01, Cohen's *d* = 1.21; shape: *t*(11) = 2.89, *p* < 0.05, Cohen's *d* = 0.83, respectively), indicating that subsequent eye movements could indeed be primed by shape or color alone.

#### Fixation analysis

**Figure 9.**
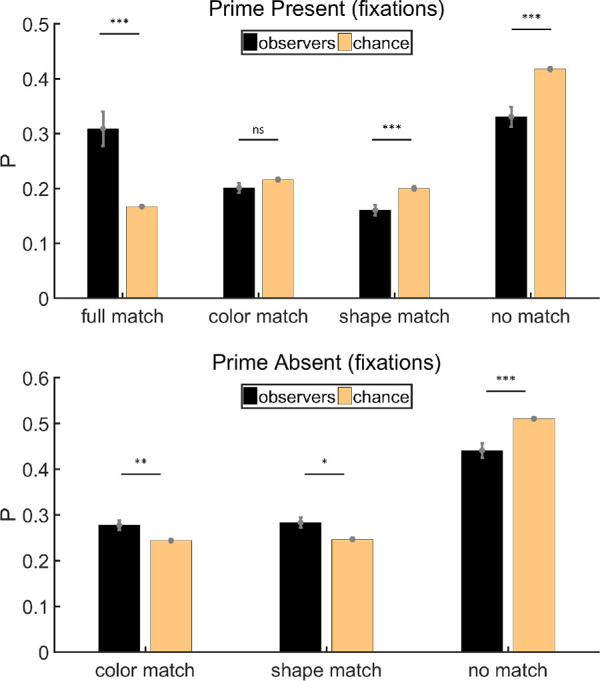
Results of fixation analysis for Experiment 2. Full match: attended item shared color and shape with the prime stimulus; color match: Items shared the same color but had different shapes; shape match: they had different colors but shared the same shape; no match: they had different colors and different shapes. Error bars are ±1 standard error. *p < 0.05. **p < 0.01. ***p < 0.001.

Trials in which we could not get any fixations were excluded before the calculation (∼3%). On both prime-present and prime-absent trials, the average number of fixations is 7. Figure 9 shows the results of fixation analysis, which are similar to the results of eye position analysis. The proportion of no match items was below chance on both prime-present and prime-absent trials (paired *t* tests, prime present: *t*(11) = −4.59, *p* < 0.001, Cohen's *d* = −1.33; prime absent: *t*(11) = −4.52, *p* < 0.001, Cohen's *d* = −1.30). More items were classified as full match on prime-present trials than chance (paired *t* test, *t*(11) = 4.83, *p* < 0.001, Cohen's *d* = 1.40). Again, on prime-absent trials, there were more fixations on color match and shape match items than predicted by chance (paired *t* tests, color: *t*(11) = 3.26, *p* < 0.01, Cohen's *d* = 0.94; shape: *t*(11) = 2.98, *p* < 0.05, Cohen's *d* = 0.86, respectively). This demonstrated that human fixation behavior was jointly primed by color and shape on prime-present trials but could be primed by color or shape alone if a perfect match was not available.

##### The *N*th fixations

**Figure 10.**
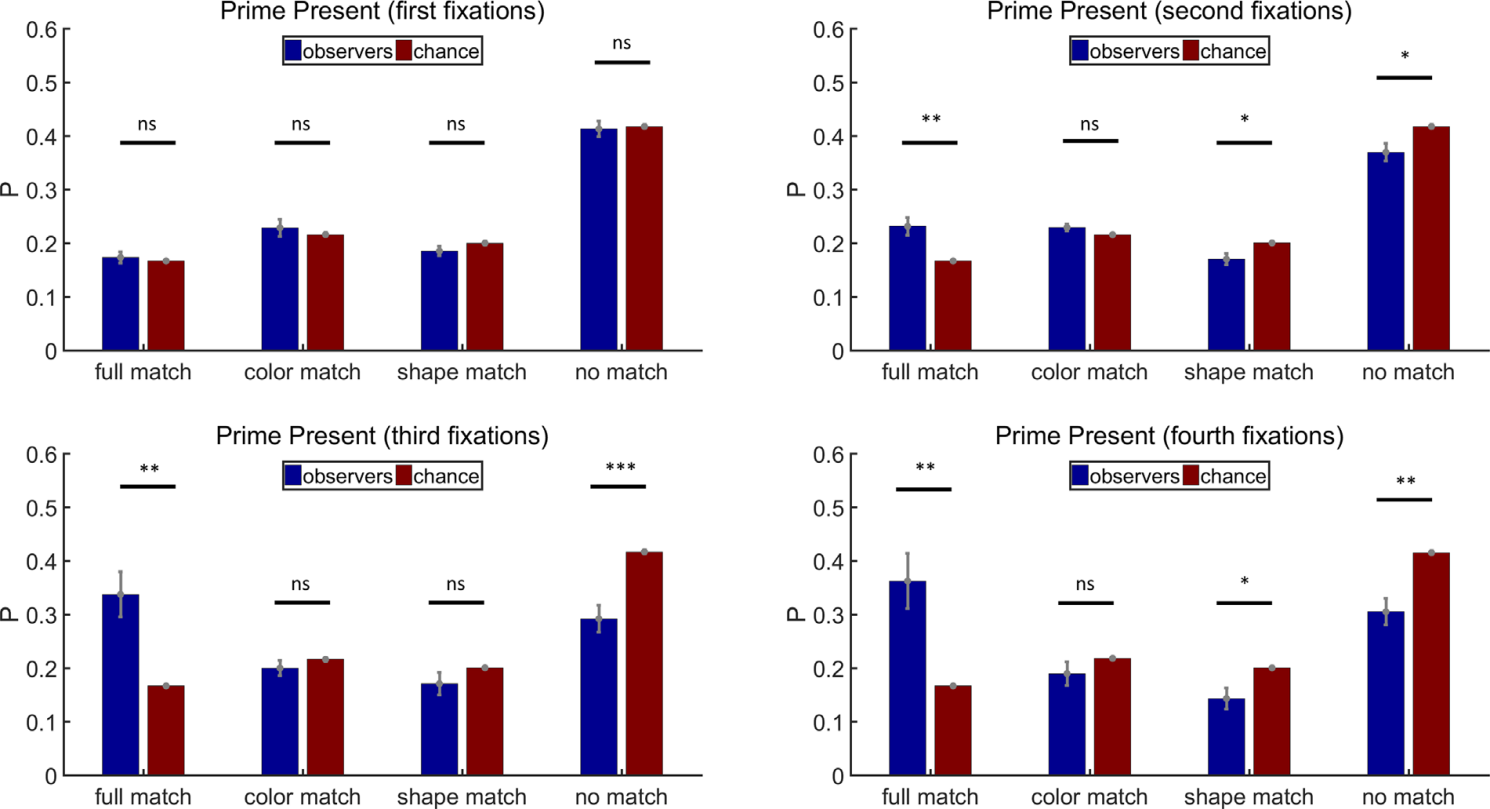
Results of the first, second, third, and fourth fixation analysis for prime-present trials in Experiment 2. Full match: attended item shared color and shape with the prime stimulus; color match: they shared the same color but had different shapes; shape match: they had different colors but shared the same shape; no match: they had different colors and different shapes. Error bars are ± 1 standard error. *p < 0.05. **p < 0.01.

Following the analysis method in Experiment 1, we also analyzed individual fixations according to their temporal order. All participants had four-fixation trials. On average, 161 trials per participant were included (max = 180, min = 103) for the analysis. Results for prime-present trials in Experiment 2 are shown in Figure 10. There was no obvious priming effect for the first fixation. The probabilities for fixations on full match, color match, shape match, or no match items all approximated chance levels (paired *t* tests, full match: *t*(11) = 0.44, *p* = 0.67, Cohen's *d* = 0.13; color match: *t*(11) = 0.64, *p* = 0.53, Cohen's *d* = 0.19; shape match: *t*(11) = −1.62, *p* = 0.13, Cohen's *d* = −0.47; no match: *t*(11) = −0.36, *p* = 0.73, Cohen's *d* = −0.10). But priming of joint features occurred persistently and strongly on the second, third, and fourth fixations (Second fixations: paired *t* tests, full match: *t*(11) = 3.91, *p* < 0.01, Cohen's *d* = 1.13; color match: *t*(11) = 1.76, *p* = 0.11, Cohen's *d* = 0.51; shape match: *t*(11) = −2.78, *p* < 0.05, Cohen's *d* = −0.80; no match: *t*(11) = −2.96, *p* < 0.05, Cohen's *d* = −0.85. Third fixations: paired *t* tests, full match: *t*(11) = 4.34, *p* < 0.01, Cohen's *d* = 1.25; color match: *t*(11) = −1.17, *p* = 0.27, Cohen's *d* = −0.34; shape match: *t*(11) = −1.76, *p* = 0.11, Cohen's *d* = −0.51; no match: *t*(11) = −5.01, *p* < 0.001, Cohen's *d* = −1.44. Fourth fixations: paired *t* tests, full match: *t*(11) = 3.97, *p* < 0.01, Cohen's *d* = 1.14; color match: *t*(11) = −1.58, *p* = 0.14, Cohen's *d* = −0.46; shape match: *t*(11) = −3.09, *p* < 0.05, Cohen's *d* = −0.89; no match: *t*(11) = −4.19, *p* < 0.01, Cohen's *d* = −1.21).

**Figure 11.**
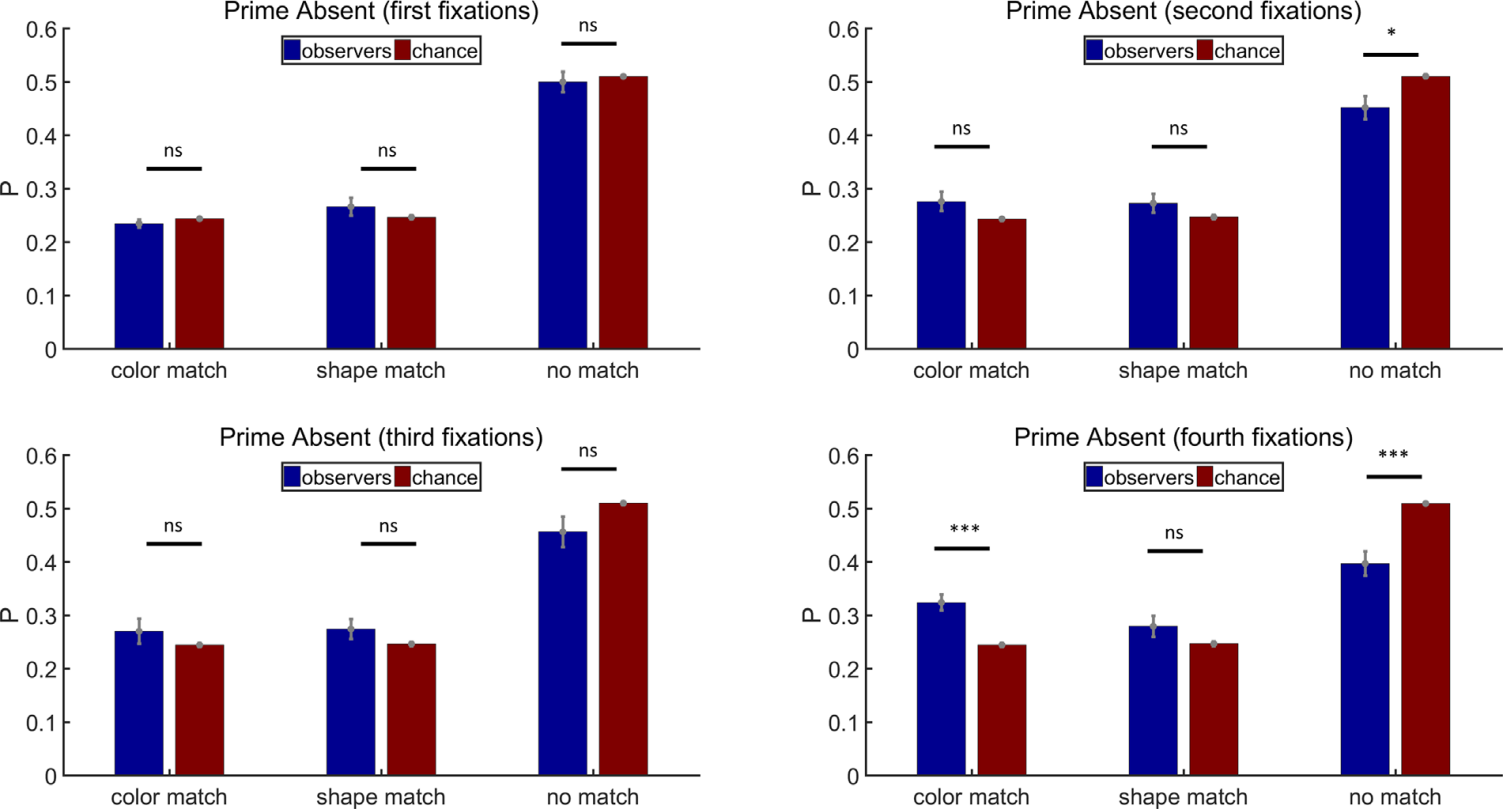
Results of the first, second, third, and fourth fixation analysis for prime-absent trials in Experiment 2. Full match: attended item shared color and shape with the prime stimulus; color match: they shared the same color but had different shapes; shape match: they had different colors but shared the same shape; no match: they had different colors and different shapes. Error bars are ± 1 standard error. *p < 0.05. ***p < 0.001.

Figure 11 shows the results of prime-absent trials in Experiment 2. Single features did not have any significant influence on the first fixations (paired *t* tests, color match: *t*(11) = −1.07, *p* = 0.31, Cohen's *d* = −0.31; shape match: *t*(11) = 1.20, *p* = 0.26, Cohen's *d* = 0.35; no match: *t*(11) = −0.56, *p* = 0.59, Cohen's *d* = −0.16). The second fixations visited no match items less often than chance predicted, but neither color match nor shape match probabilities were significantly above the chance level (paired *t* tests, color match: *t*(11) = 1.78, *p* = 0.10, Cohen's *d* = 0.52; shape match: *t*(11) = 1.37, *p* = 0.20, Cohen's *d* = 0.40; no match: *t*(11) = −2.93, *p* < 0.05, Cohen's *d* = −0.85). Nothing was significant for the third fixations, while the effects were more robust for the fourth fixations (Third fixations: paired *t* tests, color match: *t*(11) = 0.94, *p* = 0.37, Cohen's *d* = 0.27; shape match: *t*(11) = 1.55, *p* = 0.15, Cohen's *d* = 0.45; no match: *t*(11) = −1.93, *p* = 0.08, Cohen's *d* = −0.56. Fourth fixations: paired *t* tests, color match: *t*(11) = 5.47, *p* < 0.001, Cohen's *d* = 1.57; shape match: *t*(11) = 1.56, *p* = 0.15, Cohen's *d* = 0.45; no match: *t*(11) = −4.81, *p* < 0.001, Cohen's *d* = −1.39). As can be seen in Figure 11, the second, third, and fourth fixations all produced the same pattern of results. The different patterns of statistical significance reflect some underlying noisiness in the data.

#### Discussion

Experiment 2 involved a more complex change detection task. Subjects were asked to answer questions about differences in not only item number but also item color and shape. In this case, we can assume that prime features of color and shape were actively encoded into memory. The filler display was still task irrelevant. Nevertheless, eye tracking showed that eye movements were biased toward items with primed features independently in the filler display, especially on prime-present trials. As in Experiment 1, priming was weak or absent on the first fixations in Experiment 2. That first fixation on the filler may be a generic center of gravity or center of screen fixation with effects of the specific content of the filler display only appearing on the later fixations. One of the differences between Experiment 1 and Experiment 2 lies in that only the joint effect of the presence of both color and shape was significant in Experiment 1, while priming from color or shape alone was clearly demonstrated in Experiment 2. In addition, color priming appears to be somewhat stronger than shape priming in Experiment 2. The comparison between Experiments 1 and 2 suggests that the two experiments produce essentially the same results, with the magnitude of the effects being stronger in Experiment 2.

## General discussion

The study was designed to investigate whether viewing history would prime subsequent eye movements in an unusually free-viewing condition. This is not a classic inter trial priming task (Maljkovic & Nakayama, 1994, 1996, 2000) where experimenters look for the effects of one trial on the next trial. Here we are looking for effects of encoding a display on the observer's incidental eye movements while viewing a task-irrelevant filler stimulus. Our change detection task allowed us to modulate how prime was encoded. In Experiment 1, prime features (color and shape) were irrelevant to the cover task of comparing the number of items on the memory and test screens. Observers would not be explicitly motivated to consciously pay attention to color and shape prime features. However, as observers counted the number of items, color and shape features would be encoded in some fashion, just not in response to explicit instructions. Thus, in Experiment 1, we can assess if these more implicitly encoded features prime fixation behavior during the apparent filler period. In Experiment 2, on the contrary, subjects had to explicitly direct attention to the color and shape prime features in order to complete the more complex change detection task. Thus, prime features in Experiment 2 would be encoded more deeply and explicitly. Our use of the terms *implicit* and *explicit* is similar to that of Maljkovic and Nakayama (2000). Their observers were not required to report priming features in the implicit conditions, and a partial report technique was used to create their explicit conditions. One could have a narrower definition where implicit means consciously unaware. In our experiments, we are using the term in contrast to the case in which observers are given explicit instructions to engage with the color and shape of stimuli. Again, the most important feature of our design is not the change detection task. It is our freer than usual free-viewing task. There were no instructions about the filler period. Observers made no explicit responses during that period. They merely continued to have their eyes tracked and might have guessed that the filler display was intended to confuse them. It might have been valuable to ask observers what they thought about the filler task. We did not do this, though it seems unlikely that naive observers would have hypothesized that the critical measures were their eye movements during the interval between the two frames of the change detection task.

Half of the filler intervals contained a copy of the prime object while the other half did not. Thus, the two experiments crossed with prime presence/absence created four conditions of interest: (1) The prime-present trials of Experiment 1 had implicitly encoded priming features. Here, items sharing both features with the prime were favored by attention. (2) The prime-absent trials of Experiment 1 did not show reliable priming of the items that shared just one feature with the prime. But by analyzing the first through fourth fixations separately, it was found that significant color priming and shape priming appeared intermittently. (3) The prime-present trials of Experiment 2 had explicitly encoded priming features. This produced stronger bias toward attending items that shared two features with the prime and away from the items that shared no features with the prime. Attention was, if anything, also biased away from items that shared one feature with the prime, but this is probably a simple reflection of the zero-sum nature of this task. If attention was strongly attracted to matches to the prime, attention had to be drawn away from all other items. (4) This hypothesis is supported by the prime-absent trials of Experiment 2. Here, there were no items sharing both features with the prime. Consequently, items with only one common feature with the prime attracted more visual attention than items with completely different features.

All in all, the results demonstrate that explicit attention to priming features is not a prerequisite for priming but can enhance priming. The prime can automatically guide attention to stimuli with the same features as the prime in the subsequent display, no matter whether prime features were implicitly or explicitly encoded. When prime features were not explicitly encoded, priming still occurred, but to a weaker extent and only on prime-present trials, though color priming and shape priming were found intermittently during prime-absent trials. When the change detection task required explicit processing of the prime features, priming was more obvious on both prime-absent and prime-present trials.

Priming may modulate the representation of targets in the saliency maps of the frontal eye fields or superior colliculus (Fecteau & Munoz, 2003). There has been debate about whether these effects occur in the absence of some top-down intent to engage with the stimuli (Brascamp et al., 2011). Our results are in line with the position that holds that top-down intent is not required in the priming stage or the primed stage. Our result, showing that priming can be a passive automatic effect, is consistent with other studies (Theeuwes et al., 2006; Theeuwes & van der Burg, 2007, 2008, 2011, 2013).

However, passive automatic conclusion has been argued against by some researchers (Fecteau, 2007; Kristjánsson et al., 2013; Yashar et al., 2017). They argue for a goal-dependent hypothesis that predicts that repeating one target feature from the previous trial would facilitate performance only when the feature is relevant to the performed task. The studies supporting this hypothesis typically involve different search tasks in two consecutive trials, so, as discussed in the Introduction, the failure to find priming effect may be due to the interference of task switching.

Another difference between our approach and other studies is that we used eye movements as our dependent measure rather than reaction time (RT) or accuracy. Eye movements are a well-established indicator of attention shifting (Henderson & Hayes, 2018). For present purposes, they have the virtue of eliminating the need for any overt response during the free-viewing task. If our participants thought about the eye tracking at all, they probably thought it was relevant to the change detection cover task and not to the seemingly irrelevant interval between change detection displays. It is encouraging that essentially the same results were obtained by analyzing the raw eye position data or the calculated saccade and fixation data.

In summary, returning to the question about whether viewing history will influence subsequent eye movements in free-viewing conditions, the short answer is yes. Eye movements were biased toward items sharing one or more features with the prime during a free-viewing interval. Feature priming guides subsequent attention even when the primed features are encoded implicitly and even when the observers are doing nothing beyond waiting for the “real” task to continue in the subsequent display.
